# Zinc detection in oil-polluted marine environment by stripping voltammetry with mercury-free nanoporous gold electrode

**DOI:** 10.1038/s41598-022-20067-0

**Published:** 2022-09-21

**Authors:** M.-C. Clochard, O. Oral, T. L. Wade, O. Cavani, M. Castellino, L. Medina Ligiero, T. Elan

**Affiliations:** 1 Laboratoire des Solides Irradiés, CNRS-CEA-Ecole Polytechnique, UMR7642, Institut Polytechnique de Paris, 91128 Palaiseau Cedex, France; 2 Department of Applied Science and Technology (DISAT), Politecnico di Torino, C. so Duca degli Abruzzi 24, 10129 Turin, Italy; 3 TotalEnergies, PERL, Lacq, 64000 Pau, France

**Keywords:** Chemistry, Materials science, Nanoscience and technology

## Abstract

Detection of Zn(II) in oil-polluted seawater via square-wave anodic stripping voltammetry (SW-ASV) utilizing thin gold electrodes sputtered onto nanoporous poly(acrylic acid)-grafted-poly(vinylidene difluoride) (PAA-g-PVDF) membrane is herein reported. Prior to SW-ASV, PAA grafted nanopores demonstrated to efficiently trap Zn(II) ions at open circuit. This passive adsorption followed a Langmuir law. An affinity constant of 1.41 L $\upmu$mol$^{-1}$ and a maximum Zn(II) adsorbed mass q$_{max}$ of 1.21 $\upmu$mol g$^{-1}$ were found. Applied SW-ASV protocol implied an accumulation step (− 1.2 V for 120 s) followed by a stripping step (− 1.2 to 1 V; 25 Hz; step: 4 mV; amplitude: 25 mV; acetate buffer (pH 5.5)). It revealed a Zn redox potential at − 0.8 V (Ag/AgCl pseudo-reference). Multiple measurements in synthetic waters close to the composition of production waters exhibited a decreasing precision with the number of readings R (1.65$\%$ (R = 2) and 6.56$\%$ (R = 3)). These membrane-electrodes should be used as disposable. The intra-batch mean precision was 14$\%$ (n = 3) while inter-batches precision was 20$\%$ (n = 15). Linear and linear-log calibrations allow exploitation of Zn(II) concentrations ranging from 10 to 500 $\upmu$g L$^{-1}$ and 100 to 1000 $\upmu$g L$^{-1}$ respectively. The LOD was 4.2 $\upmu$g L$^{-1}$ (3S/N). Thanks to obtained calibration, a detected Zn(II) content of 1 ppm in a raw production water from North Sea oil platform was determined.

## Introduction

Since the end of 2018, OSPAR regulation for petroleum industrial waste in seawater addresses tolerable limits of toxic metals in $\upmu$g L$^{-1}$ (ppb) range (As: 60 ppb, Ni: 860 ppb, Cd: 21 ppb, Cr: 60 ppb, Cu: 260 ppb, Hg: 4.7 ppb, Pb: 130 ppb, Zn: 300 ppb). To address this issue and control the quality of wastewaters, offshore petroleum platforms need today to be equipped with portable analytical tools to monitor the metal content in production waters. These waters contains a non negligible part of oil. Due to partitioning coefficient, oil content is inversely proportional to salt content. Among the toxic metals of interest, Zn is one of the preponderant species found in petroleum production waters.

To determine metals in seawater at trace-level, electrochemical analyses such as Stripping Voltammetry (SV) have gained a lot of interest as they are portable, low-priced and may be performed in situ in salted matrices with limited interferences^1,2^. Over the last 15 years, several optimizations have been done to lower the limits of detection and to better resolve the peak current signals in case of multi-ion detection^3–5^: square wave (SW), differential pulse (DP) and vibrating modes, accumulation potential, underpotential deposition (UPD)^6^, electrode doping, electrode nanostructuration, voltammetric sensors based on nanocomposites^7–9^, chelating adsorbents agents^10–13^, etc. These analytical strategies and their combination with stripping voltammetry at solid-electrode surfaces have today reached an important reliability for trace-metal determination in a variety of sample matrices.

Using different electrode configurations and materials, anodic stripping voltammetry (ASV) was reported as an effective electroanalytical method to detect Zn(II) in natural waters^10,14–17^. In the 1990s, Tercier et al.^18^ did their measurements to in-situ monitor metal traces, comprising Zn(II), in real seawaters using Hg-based electrodes and compared DP-ASV and SW-ASV method. They concluded that the best electrochemical method in terms of reproducibility for in-situ measurements was the SW-ASV method. Up to now, mercury based electrodes (drop or film) are the most widely used as they are very accurate and efficient in preconcentrating metals. Thanks to abovementioned literature, they have exhibited a particular accuracy for Zn detection. However, they suffer important drawbacks in terms of toxicity and interferences with organic compounds, especially oil.

Very few publications indicate the use of SW-ASV with mercury-free electrodes for Zinc determination at ppb level in marine environment^1^. Among them, Gibbon-Walsh et al.^19^ reported impressive results of sub-ppb level detection of Zn(II) in seawater by ASV using a simple microwire vibrating gold electrode and exploiting the underpotential deposition method to shift Zn peak potential to more positive values. Very recently, another research team^20^ succeeded in reaching the same level of Zn(II) detection in seawater samples using a more sophisticated poly(sodium 4-styrenesulfonate)/wrinkled reduced graphene oxide composite modified glassy carbon electrode.

The aim of the present study is thus to propose another alternative for mercury-free electrodes able to detect Zn(II) in a wider range of ppb level in a mixture of petroleum and seawater. Herein electrodes are based on nanolayered gold electrodes sputtered on each side of a functionalized nanoporous poly(difluoride vinylidene) (PVDF) membrane. Very thin gold based electrodes are particularly good candidates. These electrodes have already demonstrated their efficiency in natural waters (not seawater) for lead detection^21^ and mercury detection through poly(4-vinylpyridine) grafting inside the nanoporosity of PVDF^22^. However, in order to reach the level of sensitivity in seawater required by OSPAR regulation, they need not only to be combined with an adsorptive strategy to trap and preconcentrate efficiently Zn cations but also to adapt SW-ASV protocol to enlarge the window potential to get access to negative Zn redox potential. Based on this strategy and on SW-ASV detection, poly(acrylic acid) functionalized nanoporous PVDF electrodes are herein investigated to quantitatively estimate the Zn(II) content in deionized water, synthetic water and raw production water coming from petroleum offshore production site.

## Materials and methods

### PAA-g-PVDF membrane-electrodes fabrication

(Fig. 1).

**Figure 1:**
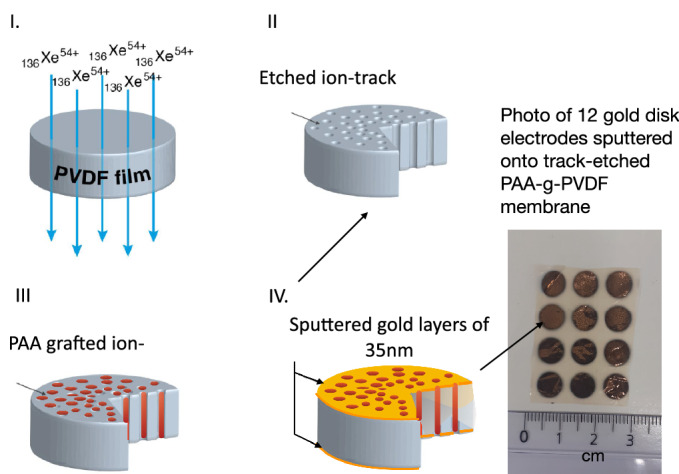
Fabrication steps of PAA-g-PVDF nanoporous membrane electrode: I. Swift heavy-ion irradiation of PVDF thin film using $_{136}$Xe$^{54+}$ ions of 5.9 MeV/mau, II. Ion-track etching, III. Radiation-induced grafting of PAA inside PVDF etched ion-tracks, IV. Sputtering of a thin gold layer (35 nm) on each side of PAA-g-PVDF nanoporous membrane. Photo of 12 nanoporous gold electrodes sputtered through a home-made mask onto PAA-g-PVDF track-etched membrane surface.

#### Swift heavy ions irradiation

Bi-oriented PVDF films were purchased from AJEDIUM (a division of Solvay Solexis, USA) (thickness: h = 9$\upmu m$). Swift Heavy Ions (SHI) irradiations were performed at GANIL, France. Films were irradiated at room temperature with Xe$^{54+}$ at energy of 5.9 MeV amu$^{-1}$ under He atmosphere. Fluence was equal to 10$^{10}$ cm$^{-2}$.

#### Track-etching

Chemical attack is performed at $65\,^{\circ }\hbox {C}$ in a solution of KOH 10 N and KMnO$_4$ 0.25 N. The final diameter depends on the etching time. To obtain a pore diameter of 50 nm, an etching time of 30 min was required. Afterwards, etched PVDF films were first washed in potassium disulfite solution (15%) and then 3 times rinsed with deionized water.

#### Radiografting and gold sputtering

Remaining radicals present in the pore walls were used to initiate a radical polymerization in presence of acrylic acid following a reported method^21^. Acrylic acid was purchased from Sigma-Aldrich without further purification. Pure liquid acrylic acid monomer solution was added. Prior the radiografting, a bubbling under nitrogen gas was done for 15 min. The radiografting reaction temperature was set at $60\,^{\circ }\hbox {C}$. Reaction time was 60 min. The resulting PAA-g-PVDF nanoporous membranes were purified for 48 h in hot deionized water thanks to a Sohxlet extraction. The membranes were dried gently at room temperature for 24 h. A gold layer of 35 nm was sputtered on both side of the nanoporous membrane through a mask to form circular electrodes of 8 mm of diameter (Fig. 1).

### Exposition of PAA-g-PVDF membrane-electrodes to tested waters (production and spiked)

1 g L$^{-1}$ stock solution of Zn(II), Pb(II) and Cu(II) were purchased from Alfa Aesar. Acetic acid (0.1 M) and NaCl were purchased from Sigma-Aldrich. For calibrations, dilutions in deionized water were realized in volumetric flasks to obtain final concentrations of 0, 100, 200, 300, 400, 500 and 1000 $\upmu$g L$^{-1}$ (ppb). At the labscale, to mimick seawater sample with high salinity content close to production waters from offshore oil drilling, repeatability and reproducibility tests have been done in synthetic waters mixed with oil. Synthetic waters were composed of 49 g of NaCl, 0.77 g of KCl, 0.11 g of CaCl$_2$:2H$_2$O, 0.20 g of MgCl$_2$:6H$_2$O, 0.01 g of Na$_2$SO$_4$ and 0.09 g of NaHCO$_3$ for 1 L. The partioning coefficient between oil and water salinity fixes the oil content. Total Organic Carbon (TOC) was 59 mg L$^{-1}$. Real production water samples coming from North Sea were provided by TotalEnergies company. For production water samples analysis, no intermediate dilution was prepared. Exposition of each membrane-electrode was performed with the same protocol for calibrations and production water analysis: one membrane-electrode was added in glass containers of 125 mL, 2/3 filled with the tested solution. The flasks were agitated for 30 min at room temperature. For each condition (concentration, water type) 3 replicates of each flasks were prepared.

### SW-ASV measurements

**Figure 2:**
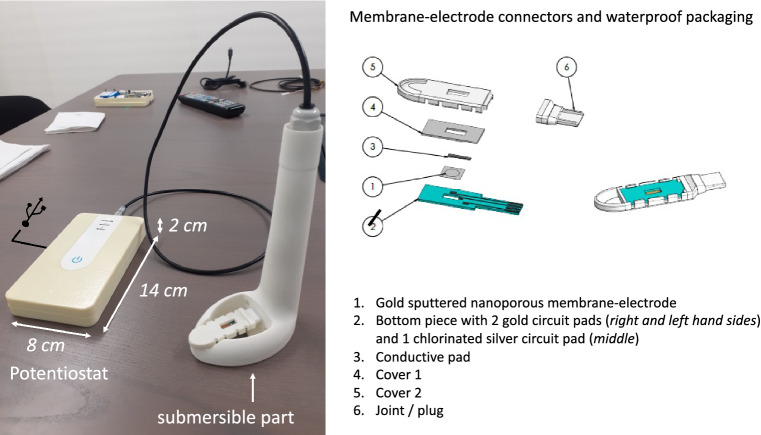
Photo of the home-made portable electrochemical sensor prototype named CAPTOT—adjacent figure: designed pieces for clipsing and connecting functionalized nanoporous membrane-electrode to the system.

A home-made portable prototype, named CAPTOT, was build (Fig. 2). It is composed of a potentiostat (developed in collaboration between LSI and VALOTEC) and a clip device (designed by IDO) ensuring a waterproof connection between the membrane-electrode gold disk and the potentiostat. Two gold circuit pads, included in the clip, allowed the contact of both membrane-electrode sides for working and counter electrodes. A chlorinated silver circuit pad was used as pseudo-reference electrode. Once the membrane-electrode pad was immersed for a certain time (10, 20, 30, 60, 120 and 240 min) in the water to analyse, an electrodeposition was then applied for 150 s. An accumulation potential of − 1.2 V allowed the reduction of trapped ions inside the porosity. The electrodeposition step was then followed by an oxidative potential scanning (stripping) from − 1.2 V to 1 V at 25 Hz in square-wave mode (4 mV step, 25 mV amplitude). The buffered electrolyte for the analysis was composed of 100 mM acetic acid and 10 mM NaCl.

### Ion chromatography

Ion chromatography measurements were performed in duplicate using Dionex EASION IC system from Thermo Electron SAS. Column: IonPac SGC 1, SCS 2, 4 mm. Concentration column: IonPac TCC, LP1, 4 $\times$ 25 mm. Eluent: 2.5 mM Methyl Sulfonic Acid (MSA)/0.8 mM oxalic acid. Temperature: $21\,^\circ$C. Flow rate: 1.0 mL min$^{-1}$. Loop volume: 500 $\upmu$L. Injection volume: 2.5 mL. Detection: non-suppressed conductivity. Calibration curve of standard Zn(II) spiked deionized water solution was plotted against conductivity peak area (see Supplementary Information) and was used for Zn(II) equilibrium concentration $c_{eq}$ determination for the adsorption study.

## Results and discussion

### Characterization of Zn trapped in PAA-g-PVDF nano-porous membranes

**Figure 3:**
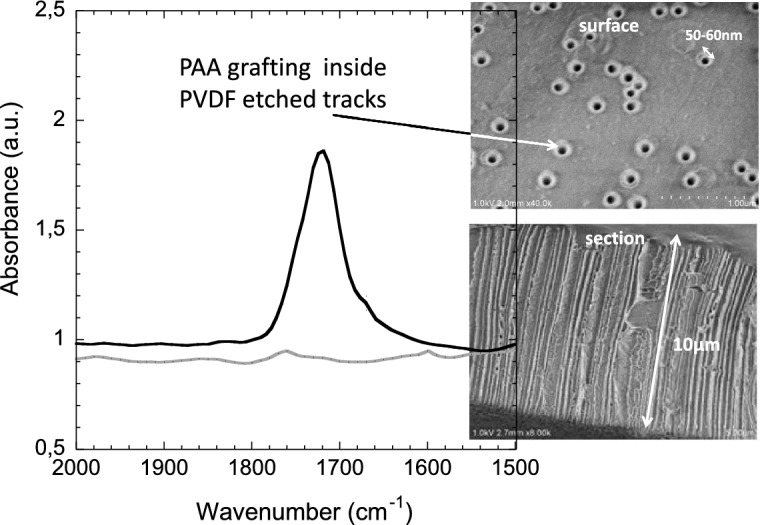
FTIR spectra of track-etched PVDF membrane after 30 min of etching in KOH/KMnO$_{4}$ solution at $65\,^{\circ }\hbox {C}$ (grey curve) and after subsequent radiation-induced grafting in presence of acrylic acid for 1 h at $60\,^{\circ }\hbox {C}$ and 48 h of Soxhlet purification (black curve)—zoom in the region of carboxylic acids between 2000 cm$^{-1}$ and 1500 cm$^{-1}$—Inset: FESEM images of PAA-g-PVDF track-etched membrane: (top) view of the surface; (down) cryosection.

The fabrication steps are summarized in Fig. 1. Once the PVDF thin films have been irradiated by swift heavy ions at the desired fluence of 10$^{10}$ cm$^{-2}$ (step I), they were chemically etched by a strong alkaline solution containing oxidative species. The ion-track etched PVDF membrane results in a membrane with well-defined cylindrical nanopores (step II). Track-etched PVDF nanopore walls are rich with radicals. These radicals have been created during ion-beam pre-treatment. Some of them were kept trapped in the crystallites of PVDF bulk with no possibility of diffusing nor recombining^2^. Freshly etched PVDF membranes with pore diameters of roughly 50 nm were then immersed in acrylic acid (AA) monomer solution. Heating the system up to $65\,^\circ$C gave sufficient energy to residual radicals to initiate the AA polymerization from the nanopore walls (step III). After irradiation, two types of radicals, alkyl and peroxyl, were present in ion-tracks. The alkyl radicals are the more reactive ones toward subsequent AA polymerization. Grafted acrylic acid functionalities are clearly evidenced by FTIR (Fig. 3).

**Figure 4:**
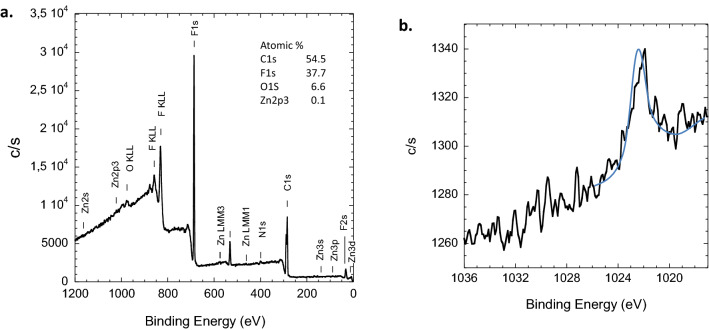
PVDF-g-PAA membrane after 30 min immersion in 500 $\upmu$g L$^{-1}$ Zn(II) spiked deionized water solution. (**a**) XPS survey spectrum; (**b**) region of Zn2p$_{3/2}$ peak at 1022.14 eV.

To evaluate the ability of PAA to trap Zn(II), PAA-g-PVDF membranes were immersed in a solution of 500 $\upmu$g L$^{-1}$ Zn(II) ions for 30 min sorption. The XPS survey spectrum predominantly exhibited the characteristic peaks of PAA-g-PVDF (Fig. 4a). After 90 min scanning, the Zn(II) signal at a binding energy of 1022 eV clearly evidenced the Zn presence (Fig. 4b). It is worth mentioning that XPS-probed surface was solely 10 nm. Most of trapped Zn(II) ions were randomly distributed all along the 10 $\upmu$m long cylindrical PAA grafted nanopores.

### Sorption of Zn(II) inside PAA grafted nanopores

**Figure 5:**
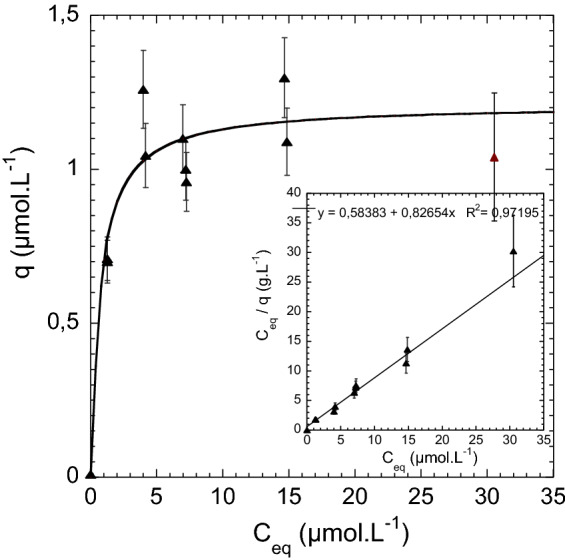
Adsorption isotherm of sorbed Zn(II) mass $\textit{q}$ versus Zn(II) equilibrium concentration c$_{eq}$.

To inform on thermodynamic equilibrium of Zn(II) complexation inside PAA grafted nanopores, sorption experiments were carried out at $21\,^\circ$C using deionized water solutions of Zn(II) with initial concentrations c$_0$ ranging from 0 to 2000 $\upmu$g L$^{-1}$ which corresponds to 0 to 30.58 $\upmu$mol L$^{-1}$. Zn(II) ions uptake by the PAA-g-PVDF nanoporous membrane has been evaluated by calculating the variation of Zn(II) concentration of the surrounded solution, c$_{eq}$ with the initial concentration, c$_{i}$. In order to be independent from our ASV developed protocol based on PAA-g-PVDF nanoporous membranes, the detection of Zn(II) ions concentrations were herein done by ion chromatography. Figure 5 displays the molar mass *q* of sorbed zinc at equilibrium per gram of membrane versus the equilibrium concentration c$_{eq}$. To fit the experimental data, a Langmuir model was expected. The Langmuir equation is expressed as follows (Eq. 1):

1 $$\begin{aligned} q= \frac{q_{max} b c_{eq}}{1 + b c_{eq}} \end{aligned}$$

where q$_{max}$ and b are the maximum sorbed mass at saturation ($\upmu$mol g$^{-1}$) and the sorption coefficient (L $\upmu$mol$^{-1}$) respectively. The linearized form of Eq. (1), known as Hanes–Woolf treatment, is written in Eq. (2).

2 $$\begin{aligned} \frac{c_{eq}}{q}= \frac{c_{eq}}{q_{max}} +\frac{1}{b q_{max}} \end{aligned}$$

When plotting experimental data as $$c_{eq}$$/q=f(c$$_{eq}$$), a linear behavior was found confirming the Langmuir formalism (inset of Fig. 5). The maximum mass q$$_{max}$$ of 1.21 $$\upmu $$mol g$$^{-1}$$ was deduced from the slope resulting in a sorption coefficient $$ {b}$$ of 1.41 (L $$\upmu $$mol$$^{-1}$$). The adsorption isotherm was then plotted to fit the experimental data. q$$_{max}$$ of sorbed Zn(II) was completely reached at 1000 $$\upmu $$g L$$^{-1}$$ (15.2 $$\upmu $$mol L$$^{-1}$$). These results witness a high affinity of PAA functionalities for Zn(II) ions while the sorption capacity of the PAA-g-PVDF nanoporous membrane for Zn(II) exhibit a micromolar per gram preconcentration regime.

### Zinc detection by square-wave anodic stripping voltammetry

PAA-g-PVDF nanoporous membranes were transformed into membrane-electrode by sputtering a thin gold layer on both membrane surfaces (Step IV of Fig. 2). The gold layer should be thin enough to not block the pore entry and thick enough to ensure a good conductivity. The nanoporous electrode is thus made of pure Au. The insulating and thick nanoporous PAA-g-PVDF polymer does not participate to the electrochemical activity. Its role is to in-situ sample metal ions from waters by trapping and pre-concentrate them inside its porosity thanks to its PAA functionalization. This adsorption step is passive (open circuit). Herein membrane-electrodes sensors have previously been studied in terms of the surface electroactivity of the nanoporous gold electrode exploiting CV data in case of lead detection^21^. It gave an electroactive area of 0.1556 cm$^{2}$. In order to enlarge the electrochemical window, 0.1 M acetate solution was buffered to pH 5.5. Then, the initial step was to register the voltammetry stripping response when spiking Zn(II) ions directly in the electrolyte without applying any accumulation potential or electrodeposition (see Supplementary Information). This step was performed to first validate the adequacy of the nature of the electrode and the electrolyte for Zn(II) detection. In a second step, accumulation potential was set. A negative accumulation potential of − 1.2 V for 150 s was needed to allow the stripping of Zn with a redox potential at − 0.8 V (chlorinated Ag pseudo-reference electrode).

**Figure 6:**
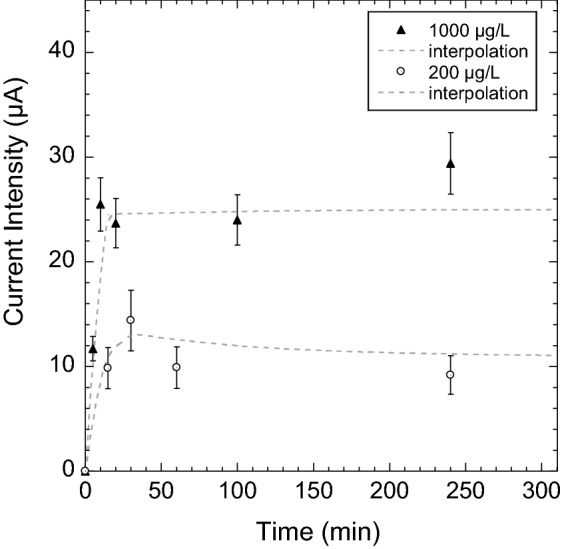
Kinetics of adsorption of Zn(II) inside PAA-g-PVDF nanoporous membrane-electrodes at 200 $\upmu$g L$^{-1}$ and 1000 $\upmu$g L$^{-1}$ in deionized water. SW-ASV detection parameters: step 4 mV, amplitude 25 mV, accumulation potential of − 1.2 V for 150 s, scan rate of 25 Hz from − 1.2 to 1 V, electrolyte: 0.1 M sodium acetate.

An important parameter to fix was the time for sorbed Zn(II) to reach equilibrium inside membrane-electrodes. The adsorption of Zn(II) ions by complexation with PAA functionalities is herein passive (open circuit). Kinetics of adsorption are displayed in Fig. 6. The equilibrium is reached after 30 min.

**Figure 7:**
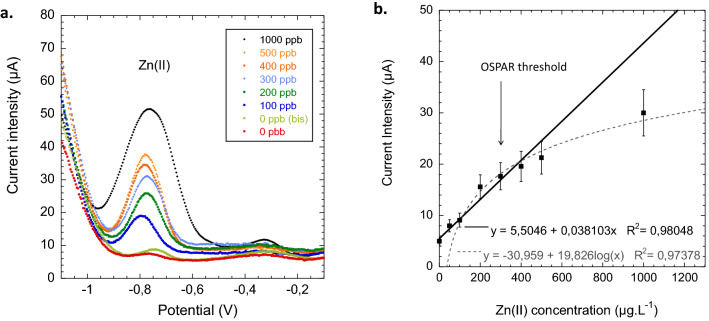
(**a**) SW-ASV voltammograms of Zn(II) obtained with PAA-g-PVDF nanoporous membrane-electrodes after a preconcentration step by adsorption of 30 min in standard Zn(II) water solutions and subsequent measurement in 0.1 M buffered acetate, pH 5; (**b**) Obtained calibration curve in the extended concentration range of 10–1000 $\upmu$g L$^{-1}$ (ppb) around OSPAR threshold for Zn in the case of production water from offshore petroleum platform.

During the electrodeposition step, part of trapped sorbed Zn(II) ions migrated to the working electrode where they were reduced into Zn(0). Then, a potential scan from − 1.2 to + 1 V permitted to reversely oxidize from Zn(0) atoms to Zn(II) ions when reaching Zn(0/II) redox potential at − 0.8 V as shown in Fig. 7. The peak for Zn is close to the base of the hydrogen wave. Such a proximity leads to a non linear baseline in this region and makes difficult to quantify Zn using SW-ASV at very low concentrations. Alternative techniques to SW-ASV did not give better signal to noise ratio as previously reported for Zn(II) detection in seawaters comparing with Differential Pulse Voltammetry (DPV)^18^ or Anodic Stripping Chronopotentiommetry (ASC)^19^.

Each point of calibration curve displayed on Fig. 7b represents the mean value of Zn(II) peak heights (triplicate experiments n = 3) at − 0.8 V. Taking the peak heights instead of the areas to plot the calibration curve permitted to exploit a wider linear range response (up to 500 mg L$^{-1}$) with a good linear regression coefficient and facilitate results implementation in the software of CAPTOT potentiostat (co-development with VALOTEC company—see Materials and methods). The linear part of the calibration curve was in the range of 10–500 $\upmu$g L$^{-1}$ while the linear-log fit allows to exploit a wider range of 100–1000 $\upmu$g L$^{-1}$. It is important to note the adequacy of concentration range with industrial need (OSPAR tolerable limit is 300 ppb for Zn content in production waters). The mean error of each set of measurements was 15$\%$ (n = 3) and 20$\%$ (n = 15 triplicate experiment of 5 inter-batches membrane-electrodes). It was also observed that a ping-pong stirring, set at 200–250 rpm equivalent, did not give better results than rotating stirring set at 300 rpm. The Limit of Detection (LOD) was determined from three times the standard deviation of blank experiments and was found equal to 4.2 $\upmu$g L$^{-1}$.

### Repeatability, reusability and selectivity in synthetic waters

**Figure 8:**
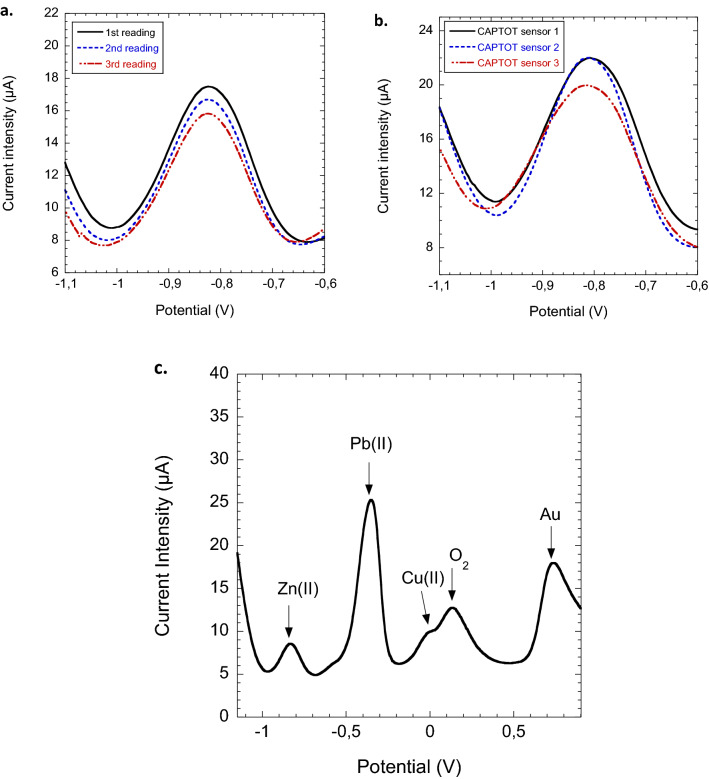
(**a**) Repeatability test: multiple SW-ASV voltammograms registrations obtained with the same presorbed Zn(II) PAA-g-PVDF nanoporous membrane-electrode; (**b**) reproducibility test: triplicate experiments utilizing three different batches of PAA-g-PVDF nanoporous membrane-electrodes—for (**a**) and (**b**) tests, presorptions were performed in 200 ppb Zn(II) spiked synthetic water solutions; (**c**) multiple-ion detection: registered SW-ASV voltammogram of a presorbed PAA-g-PVDF nanoporous membrane-electrode in an aqueous solution composed of 50 g L$^{-1}$ NaCl, 200 ppb Zn(II), 200 ppb Pb(II) and 200 ppb Cu(II)—Electrolyte: 0.1 M buffered acetate, pH 5.5.

Neutral pH synthetic waters mimicking an oil polluted seawater environment containing 50 g L$^{-1}$ of various salts and 59 mg L$^{-1}$ of TOC (see Materials and methods) were used for repeatability tests (Fig. 8a,b). No significant Zn signal interference was caused either with TOC content or with addition of 0.80 M NaCl, 0.80 mM KCl, 0.70 mM Ca$^{2+}$, 0.98 mM Mg$^{2+}$, 0.07 mM Na$_2$SO$_4$ and 1.04 mM NaHCO$_3$. Multiple measurements with the same nanoporous membrane-electrode exhibited a decreasing precision with the number of readings R (1.65$\%$ (R = 2) and 6.56$\%$ (R = 3)) due to the constant diminution of trapped Zn content in the pores after each SW-ASV reading (Fig. 8a). These membrane-electrodes should only be used as disposable (one measurement per membrane). Similarly to calibration data obtained in deionized water, triplicate experiment measurements gave a mean precision of 14$\%$ (n = 3) (Fig. 8b). These results suggest that the variation is mainly due to membrane-electrode fabrication. It is worth mentioning that no special storage is needed for these sensors. They can be used several months after their fabrication without additional deviation on the measurement.

Functionalized PAA-g-PVDF nanoporous membrane complexes all metal cations by simple electrostatic interaction. This adsorption makes PAA-g-PVDF membrane selective for metals but not ion specific. PAA-g-PVDF membrane-electrode can be used to simultaneously screen multiple ions by simple in-situ sampling. Figure 8c displays a multiple ion detection in metal spiked 50 g L$^{-1}$ NaCl solution. The simultaneous addition of 200 $\upmu$g L$^{-1}$ (0.96 $\upmu$M) Pb, 200 $\upmu$g L$^{-1}$ (3.14 $\upmu$M) Cu and 200 $\upmu$g L$^{-1}$ (3.06 $\upmu$M) Zn spiked NaCl solution did not interfere with the redox peak position but strongly decreased the Zn signal by 50$\%$. It has been shown that, when the concentration of Cu and Zn are similar, as in this work, no suppression of peak current is observed^18^. The interference should consequently come from Pb presence. This electrode saturation reflects a competition of metal ions on PAA complexing sites during passive adsorption step. It is worth mentioning that all these metal additions were at least 1000 times greater than natural levels. Nevertheless, in the framework of industrial wastewaters, high levels of metal pollutants can be reached, notably in case of malfunction at offshore platform. It is thus important for the sensor to continue to alert on the pollution event under severe conditions. This peak signal reduction phenomenon can be taken into account numerically in the developed software of CAPTOT prototype.

Under our conditions, dissolved oxygen was not found to interfere with the voltammetry of Zn as its large background peak current centered at + 0.25 V was far from the Zn signal. It could be noticed that it did have an effect on Cu signal, hindering part of it (Fig. 8c). Acetate buffer electrolyte can thus be used without the need to purge with nitrogen before analysis.

### SW-ASV Zn(II) measurements in real production water

**Figure 9:**
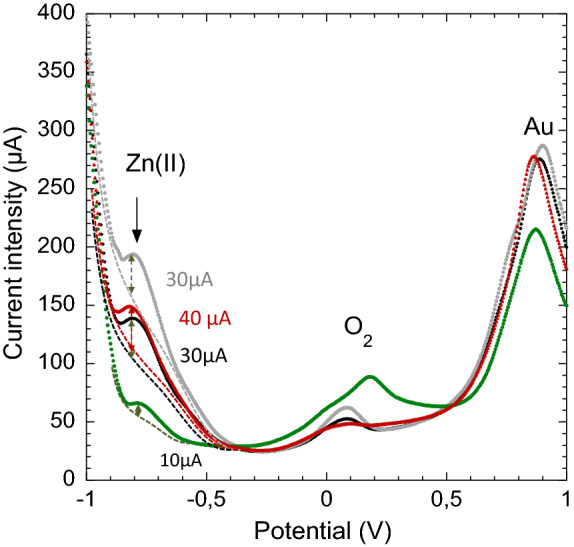
Square-wave anodic stripping voltammograms obtained using a PAA-g-PVDF membrane-electrode in 80 mL of: (1) raw production waters (grey and black curves—duplicate experiments); (2) a presorbed production water which was already in contact for 30 min with a PAA-g-PVDF membrane-electrode (green curve); (3) a 1 ppm Zn(II) spiked presorbed production water (red curve).

Samplings of raw production water coming from offshore petroleum platform in the North Sea have been put in glass containers. SW-ASV measurements were done just after immersing PAA-g-PVDF nanoporous membrane-electrodes, one per container, directly inside the production waters without any post-treatment (grey and black curves of Fig. 9).

The first observation is that the hydrogen peak at − 1.2 V was shifted to − 1 V in the presence of raw production water. This is due to a slightly acidic environment. A peak at − 0.8 V corresponding to Zn(0/II) redox potential appeared on the slope of the hydrogen peak. The main metallic pollution in this raw production water was thus attributed to zinc content. ICP-MS measurement of these raw production samples could not give a precise concentration value of zinc due to the complexity of these oil-polluted seawater matrices which can reach up to 50 g L$^{-1}$ of NaCl content. It indicated a possible zinc presence up to 1 ppm, a limit of detection not compatible with OSPAR regulation (see Supplementary Information).

Zn(II) peak heights were estimated from the tangent of the hydrogen peak slope and a reproducible 30 $\upmu$A signal was registered in duplicate measurements. From calibration curve (Fig. 6b), it corresponds to 1000 $\upmu$g L$^{-1}$ or 1 ppm. Production water samples containers which were in contact with a PAA-g-PVDF membrane (1 cm$^{2}$) for 30 min, have been measured once again by SW-ASV. It resulted in a drastic diminution of Zn(II) peak at − 0.8 V (green curve of Fig. 9). This result shows that the membrane-electrode sorption capacity was good enough to significantly lower Zn(II) initial concentration of 1 ppm to hundreds of ppb. These presorbed raw production water samples were then spiked by 1 ppm Zn(II) stock solutions and were measured subsequently by SW-ASV (red curve of Fig. 9). The initial SW-ASV profile of raw production water was totally recovered with a slight increase of 10 $\upmu$A (40 $\upmu$A instead of 30 $\upmu$A) which may come from residual Zn(II) in the solution. No change of voltammogram profile was observed confirming Zn(II) attribution. A Zn(II) content of 1 ppm in this raw production water could consequently be estimated.

### Comparison with Zn(II) electrochemical sensors for true application in seawaters

Very few research teams have developed a Zn(II) electrochemical sensor demonstrating true or high potential application for real seawater analyses (Table 1). Many electrodes suffer from strong interferences with NaCl saline, biomass and oxygen content. As shown in Table 1, the SW mode is the most commonly used solution for in-situ applications as it strongly helps in minimizing the drift of registered current baseline during the stripping and potential scanning. To below the limit of sensitivity for trace metal in such complex seawater matrices, the best electrodes up to now are mercury-based electrodes. Among mercury-free electrodes which have demonstrated high potential for Zn detection in real seawater samples, our system is less sensitive by an order of magnitude. Apart playing on electrode composition, a simple parameter to play on would be to increase the deposition time as done by other teams. However, one should know that increasing the deposition time decreases the concentration range as the electrode saturation appears earlier. In the context of industrial production waters, the sensors should perform in hundreds of ppb level and not be restricted to trace level. New advances in Screen-Printed Electrodes (SPE)^23^ may offer such a possibility in terms of concentration range with similar sensitivity with the proposed sensors. However, SPEs have not yet been reported to function in real seawater samples. The key feature of the proposed nanoporous gold electrodes lies in the supportive functionalized nanoporous polymer which allows Zn(II) in-situ sampling directly in oil polluted seawaters. Because the sampling step is made at zero current and SW-ASV analysis is done ex-situ, many artefacts are herein eliminated. Besides, another source of signal perturbation may come from oil presence. None of these techniques were confronted with the presence of such high TOC content (several tenths of mg L$^{-1}$) as this work.

**Table 1:** Comparison of Zn(II) electrochemical sensors demonstrating true or high potential application for real seawater analyses with reported sensor—for data conversion molar concentrations were multiplied by $M_{Zn}$ = 65.39 g mol$^{-1}$.

Electrode	Technique—in-situ or on-site solution	Deposition time	Linear range ($\upmu$g L$^{-1}$ )	LOD ($\upmu$g L$^{-1}$)	References
Hg based electrode	SWASV—voltammetric in-situ submersible profiler	300 s at − 0.75 V and 180 s at − 0.35 V	0.1–10	0.002	^18,24^
Vibrating gold microwire	SWASV—ex-situ measurements with in-situ potentiality	300 s at − 0.9 V	0.065–6.5	0.02	^19^
Liquid crystal Bismuth film	SWASV—flexible sensors array attached to autonomous kayak	180 s at − 1.6 V	0.3–70	0.08	^25^
Poly(sodium 4-styrenesulfonate) wrinkled rGO composite	DPASV—ex-situ measurements	300 s at − 1.4 V	0.3–50	0.1	^20^
Thin nanoporous gold disk sputtered onto PAA-g-PVDF membranes	SWASV—on-site solution with submersible probe for in-situ sampling	150 s at − 1.2 V	10–500 (100–1000 linear-log fitting)	4.2	This work

## Conclusions

Functionalized PAA-g-PVDF nanoporous membrane-electrodes of 10$^{10}$ nanopores per cm$^{2}$ have exhibited a good sensitivity for Zn(II) detection by SW-ASV in deionized, salted and petroleum production waters covering a large range of concentration from 10 $\upmu$g L$^{-1}$ to few mg L$^{-1}$. Zn(II) ions uptake by PAA functionalities were fully characterized not only by SW-ASV but also by XPS and sorption experiments using ion chromatography. Zn(II) adsorption equilibrium was reached within 30 min of adsorption. A high affinity of PAA functionalities for Zn(II) ions in deionized water was found (*b* = 1.41 L $\upmu$mol$^{-1}$) and a maximum Zn(II) sorption capacity of the PAA-g-PVDF nanoporous membrane of 1.21 $\upmu$mol g$^{-1}$ was completely reached for equilibrium concentrations equal 1000 $\upmu$g L$^{-1}$. Zn(II) micromolar per gram of membrane preconcentration regime was found of higher rate in seawater matrices environment as shown by SW-ASV measurements of real production waters after two subsequent membrane-electrodes sorptions, lowering Zn(II) initial concentration of 1 ppm to hundreds of ppb.

The limit of detection was estimated to be 4.2 $\upmu$g L$^{-1}$ (ppb) while the uncertainty in slightly acidic production waters increases by at least a factor two due to the hydrogen peak shift. One may expect more accuracy. However, the present portable technique gives sufficient reliability, well below OSPAR regulation limits of 300 ppb for Zn. This technique allows on-site Zn monitoring, directly in the glass containers, just after production water sampling.

It is also interesting to note that, after Zn(II) spiking, the use of PAA-g-PVDF membrane-electrodes coupled with SW-ASV sensoring have shown a good Zn recovery in production water. This result suggests that interferences were rather limited despite the high salt content and oil presence.

## Supplementary Information

Supplementary Information.

## Data Availability

All data generated or analysed during this study are included in this published article (and its Supplementary Information file).
