# Supplementary material for: Zinc detection in oil-polluted marine environment by stripping voltammetry with mercury-free nanoporous gold electrode
Source: Sci Rep. 2022 Sep 21;12:15771. doi: 10.1038/s41598-022-20067-0 (PMC9492727; doi:10.1038/s41598-022-20067-0)
Supplement: Supplementary file 1 — Supplementary Information. [file 41598_2022_20067_MOESM1_ESM.pdf]

1 Supplementary Information

1.1 Direct SW-ASV measurements in spiked Zn(II) waters without adsorption preconcentration

Figure 1 displays obtained results of SW-ASV measurements performed using PAA-g-PVDF membrane-electrodes of 10^{10} nanopores per cm^{-2} without adsorption prior electrochemical run.

SW-ASV parameters: electrodeposition time of 150 s at -1.2 V, potential scanning from -1.2 V to 1 V, 25 Hz, Step 4mV, amplitude 25 mV, 0.1 M buffered acetate.

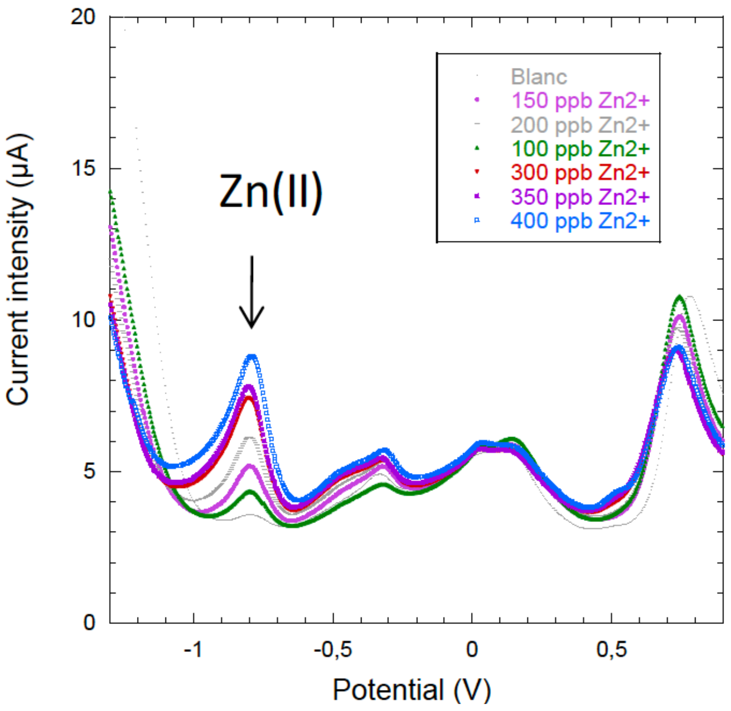

Figure 1: a. SW-ASV voltammograms of Zn(II) obtained with PAA-g-PVDF nanoporous membrane-electrodes in Zn(II) spiked deionized water of various concentrations: 100, 150, 200, 300 and 400 μg.L^{-1} in 0.1 M buffered acetate, pH 5

This step was performed to test the efficiency of chosen electrolyte and the 35 nm gold layer electrodes sputtered on each side of PAA-g-PVDF nanoporous membrane. As expected, current intensities were herein lower than in case of adsorption preconcentration.

1.2 ICP-MS production water

			Métaux
LS440 : **Lithium (Li)**	mg/l		7.01
LS3NB : **Bore (B)**	mg/l		36.1
LS2WG : **Calcium (Ca)**	mg/l		6710
LS2WH : **Fer (Fe)**	mg/l	*	23.7 ±5.93
LS2WF : **Magnésium (Mg)**	mg/l	*	1430 ±429
LS3B1 : **Potassium (K)**	mg/l		692
LS2TJ : **Aluminium (Al)**	µg/l		280
LS2NC : **Arsenic (As)**	µg/l	*	<50
LS2TA : **Baryum (Ba)**	µg/l	*	170000 ±34000
LS9H6 : **Béryllium (Be)**	µg/l		<50
LS2NE : **Cadmium (Cd)**	µg/l	*	<2.0
LS2TB : **Chrome (Cr)**	µg/l	*	12 ±4
LS2TF : **Cobalt (Co)**	µg/l	*	<10
LS2TK : **Cuivre (Cu)**	µg/l	*	13 ±3
LSMZS : **Mercure (Hg)**	µg/l		<0.015
LS2TG : **Molybdène (Mo)**	µg/l	*	<10
LS2TC : **Nickel (Ni)**	µg/l	*	10 ±3
LS2ND : **Plomb (Pb)**	µg/l	*	<10
LS2TD : **Vanadium (V)**	µg/l	*	<10
LS2TM : **Zinc (Zn)**	µg/l		<1000

Figure 2: List of metallic cations detected by ICP-MS in studied production water samples
